# Ethylene biosynthesis in legumes: gene identification and expression during early symbiotic stages

**DOI:** 10.1093/jxb/eraf069

**Published:** 2025-02-19

**Authors:** Germán O Gómez-Fernández, Robin van Velzen, Jeong-Hwan Mun, Douglas R Cook, Wouter Kohlen, Estíbaliz Larrainzar

**Affiliations:** Institute for Multidisciplinary Research in Applied Biology (IMAB), Universidad Pública de Navarra (UPNA), Campus de Arrosadía, Pamplona, E-31006, Spain; Biosystematics Group, Department of Plant Sciences, Wageningen University and Research, 6708 PB Wageningen, The Netherlands; Department of Bioscience and Bioinformatics, Myongji University, Yongin, 17058, South Korea; Department of Plant Pathology, University of California, Davis, CA 95616, USA; Laboratory of Cell and Developmental Biology, Department of Plant Sciences, Wageningen University and Research, 6708 PB Wageningen, The Netherlands; Institute for Multidisciplinary Research in Applied Biology (IMAB), Universidad Pública de Navarra (UPNA), Campus de Arrosadía, Pamplona, E-31006, Spain; Istituto di Biologia e Biotecnologia Agraria (IBBA) – CNR, Italy

**Keywords:** ACC oxidase, ACC synthase, hormones, nitrogen fixation, nodules, symbiosis

## Abstract

The final steps of ethylene biosynthesis involve the consecutive activity of two enzymes, 1-aminocyclopropane-1-carboxylate synthase (ACS) and 1-aminocyclopropane-1-carboxylate oxidase (ACO). These enzymes are encoded by small gene families, which, in the case of legumes, have not been systematically characterized at the level of gene family membership or phylogenetic relationship. Moreover, the absence of consensus nomenclature complicates comparisons within the scientific literature, where authors are addressing the roles of these genes *in planta*. In this study, we provide a framework in which the *ACS* and *ACO* gene family members of several legume species, including the two model legumes *Medicago truncatula* and *Lotus japonicus*, were systematically annotated, named, and analysed relative to genes from other dicot and monocot model species. A combination of phylogenetic and reciprocal BLAST analyses was used to identify evolutionary relationships among genes, including the identification of orthologous relationships that can inform hypotheses about function. Given the role of ethylene as a negative regulator of the legume-rhizobium symbiosis, we queried publicly available RNA-seq expression datasets to obtain an overview of the expression profiles of these genes in the interaction between *M. truncatula* and its nitrogen-fixing microsymbiont. The resulting evolutionary framework, as well as structural and expression analyses, are intended to facilitate ongoing functional studies in legumes.

## Teaser

The identity and nomenclature of members of the 1-aminocyclopropane 1-carboxylic acid (ACC) synthase and oxidase gene families in legumes remain ambiguous. Here, we provide a curated list of genes, resolve their phylogenetic relationships, and analyse their expression in roots in symbiosis with nitrogen-fixing bacteria.

## Introduction

Since its discovery in the early twentieth century (Neljubov, 1901), the gaseous phytohormone ethylene (C_2_H_4_) has been shown to act as a key regulator of numerous developmental and physiological processes in plants, ranging from seed germination to senescence and stress responses (Bakshi *et al.*, 2015; Binder, 2020; Li *et al.*, 2022). Ethylene is synthesized from methionine via a biosynthetic pathway known as the ‘Yang’ cycle (Adams and Yang, 1979; Boller *et al.*, 1979). In this cycle, *S*-adenosyl methionine is converted to 1-aminocyclopropane 1-carboxylic acid (ACC) by ACC synthase (ACS) enzymes. ACC is then converted to ethylene, CO_2_, and cyanide by ACC oxidase (ACO; Hamilton *et al.*, 1991; Ververidis and John, 1991).

The study of ethylene in legumes is particularly relevant given the important role that this hormone plays in the interaction of the host plant with symbiotic rhizobium bacteria (Penmetsa and Cook, 1997; Guinel and Geil, 2002). During this symbiosis, there is a complex molecular dialogue between the plant and the bacteria that culminates in the formation of a new organ in the root, the nodule. Within the nodule, bacteria differentiate into bacteroids in infected cells, their nitrogen-fixing form, establishing a beneficial symbiosis for both partners: bacteroids receive carbon in the form of malate, whereas the plant benefits from a source of reduced nitrogen in the form of ammonia. Ethylene is a well-known negative regulator of symbiotic development, affecting several key processes, from early signalling events to nodule establishment (Desbrosses *et al*., 2011; Guinel, 2015; Velandia *et al*., 2022). One of the first observations of the negative effects of ethylene on nodulation was reported by Grobbelaar *et al.* (1971), where the application of exogenous ethylene to rhizobium-inoculated bean roots led to fewer nodules and lower nitrogen fixation rates. Subsequently, the inhibitory effect of exogenous ethylene on nodulation has been demonstrated in other legume species, including pea (*Pisum sativum* L.; Drennan and Norton, 1972; Lee and LaRue, 1992), white clover (*Trifolium repens* L.; Goodlass and Smith, 1979), vetch (*Vicia sativa* L.; van Spronsen *et al.*, 1995), and alfalfa (*Medicago sativa* L.; Peters and Crist-Estes, 1989), among others. Although exogenous ethylene can have similar effects on soybean (*Glycine max* (L.) Merr.), variability was observed depending on the cultivar tested (Xie *et al.*, 1996). Interestingly, soybean ethylene-insensitive *etr1-1* mutants form a similar number of nodules to the wild type (Schmidt *et al*., 1999). However, the polyploid nature of the soybean genome provides opportunities for sub-functionalization that may confound interpretation of single gene mutants. Nevertheless, generalizations about a negative role for ethylene in the nodulation of all legumes may be inappropriate. For example, in the case of the semi-aquatic legume *Sesbania rostrata* Bremek. & Oberm., nodulation via intercellular invasion on stems and submerged roots requires ethylene (D’Haeze *et al*., 2003), while root hair infection under non-flooded conditions is inhibited by ethylene (Goormachtig *et al*., 2004).

The identification of ethylene-insensitive mutants in *Medicago truncatula* Gaertn. provided one the first pieces of evidence of a role for the endogenous hormone in the nitrogen-fixing symbiosis. In *M. truncatula*, the *sickle* (*skl*) mutant was identified in a genetic screen due to its hyper-infected and hyper-nodulating phenotype (Penmetsa and Cook, 1997). Subsequent efforts (Penmetsa *et al.*, 2008), revealed that *skl* is the *Medicago* orthologue of *Arabidopsis thaliana ETHYLENE INSENSITIVE2 (EIN2)*, a key component of ethylene signalling in plants (Alonso *et al*., 1999). In *Lotus japonicus* (Regel) K. Larsen, this gene is duplicated (Chan *et al*., 2013; Miyata *et al.*, 2013) and, as such, only the double mutant *Ljein2-1 ein2-2* shows an ethylene-insensitive phenotype, including hyper-nodulation (Reid *et al.*, 2018). Because the last common ancestor of *Lotus* and *Medicago* pre-dates the warm season/cool season legume divide, the negative role for ethylene in nodulation likely also pre-dates the origin of the majority of legume crops, including soybean. Ethylene is a sensitive modulator of nodulation (Nod) factor signalling, affecting Nod factor-induced calcium spiking (Oldroyd *et al.*, 2001). Additionally, it serves as a negative regulator of rhizobial infection and determines the spatial nodule positioning in the roots (Heidstra *et al.*, 1997; Penmetsa and Cook, 1997; Nukui *et al.*, 2000, 2004; Prayitno and Mathesius, 2010). Several reports have described a burst of ethylene production during early symbiotic nodulation stages, in both determinate and indeterminate legumes (Ligero *et al.*, 1987; Suganuma *et al.*, 1995; Lopez-Gomez *et al.*, 2012; Reid *et al*., 2018). In *V. sativa*, this overproduction of ethylene upon inoculation with compatible rhizobium produced a root phenotype with thick short roots (*tsr*) and nodules at unusual locations (Zaat *et al.*, 1989; van Spronsen *et al.*, 1995). Subsequent studies were able to associate this burst in ethylene production with the transcriptional induction of several ethylene biosynthesis genes. Indeed, multiple *ACS* genes in *M. truncatula* (Larrainzar *et al.*, 2015; van Zeijl *et al.*, 2015) and an *ACO* gene in *L. japonicus* (Miyata *et al.*, 2013) are induced in roots in a Nod factor dependent manner. Interestingly, ethylene production is not associated with a general defence response but is dependent on Nod factor recognition.

ACS and ACO proteins are typically encoded by small gene families (usually five to seven members; Booker and DeLong, 2015; Houben and Van de Poel, 2019). Best characterized in Arabidopsis and tomato, the proliferation of paralogous enzymes at both steps in ethylene biosynthesis has led to a complex layering of biosynthetic control, including diverse regulatory interactions and a range of tissue and situational specificities (Pattyn *et al.*, 2020). Multigene families are also found in legumes, but their precise relationships remain uncertain. Thus, to better understand the role of ethylene in nodulation, it is necessary to characterize *ACS* and *ACO* gene families in greater detail.

In the model legume *M. truncatula*, early versions of the genome documented unexpectedly large gene families, with 13 or 14 predicted *ACS* genes in Mtv3.5 (Young *et al.*, 2011) and Mtv4.0 (Tang *et al.*, 2014), respectively, and a similar situation with *ACO* gene paralogues (156 predicted genes in Mtv3.5 and 46 predicted genes in Mtv4.0). In the more recent and complete *M. truncatula* genome (Mtv5.0; Pecrix *et al.*, 2018), the number of annotated genes decreased to 10 *ACS* and 14 *ACO* gene family members. Nevertheless, a systematic study of these gene families in *M. truncatula* has not yet been performed and gene nomenclature used in the literature lacks standardization, which further complicates data interpretation and comparisons among studies. This lack of systematic identification of *ACS* and *ACO* members is true in other legumes as well, including the model species *L. japonicus* and crops such as chickpea (*Cicer arietinum* L.) and peanut (*Arachis duranensis* Krapov. & W.C.Greg.).

The aim of this review is to systematically identify and define members of the *ACS* and *ACO* gene families in the model legumes *M. truncatula* and *L. japonicus*, as well as in crop legume species selected to represent key evolutionary nodes, i.e. chickpea at the base of the warm season legumes and peanut at the base of the Papilionoidea sub-family. Genes were identified and validated using reciprocal-BLAST and phylogenetic approaches, leading to a proposed standard gene identification and nomenclature for the research community. Furthermore, we consider the expression patterns of the *ACS* and *ACO* gene families, using data obtained from publicly available RNA-seq and cell-type specific expression datasets in *M. truncatula*, with an emphasis on the early stages of rhizobium infection in roots. This work establishes a basis for future functional studies of the legume-rhizobium symbiosis.

### Identification and nomenclature of *ACS* and *ACO* genes in selected legume genomes

To identify members of the *ACS* and *ACO* gene families in legumes, we performed a reciprocal best hits BLAST search using functionally verified protein sequences as queries (Houben and Van de Poel, 2019). We used the well-characterized ACS family in *Arabidopsis thaliana* (L.) Heynh. as a reference (Rodrigues-Pousada *et al.*, 1993; Yamagami *et al.*, 2003) and searched for orthologues in a selection of legume genomes. We selected the two most common model legumes for nodulation studies: *M. truncatula*, including ecotypes Jemalong A17 and R108, and *L. japonicus* Gifu and MG-20. Legume crops with different nodulation types were also included. Two species from the genera *Arachis* were selected, including *A. duranensis* and *Arachis ipaensis* Krapov. & W.C.Greg. which are the diploid ancestors of cultivated peanut (*Arachis hypogaea* L.; Bertioli *et al.*, 2016). *Arachis* species are near the base of Papilionoid legumes, with determinate nodulation and an atypical ‘crack entry’ infection (Horta Araújo *et al.*, 2024). We also selected three genomes of *Cicer*, including cultivated chickpea (*Cicer arietinum* L. ICCV 96029) and its immediate wild crop relatives (*Cicer echinospermum* Gunasan and *Cicer reticulatum* Besevler) as examples of warm season legumes with indeterminate nodulation. The use of multiple independent genomes in each of four legume genera (*Medicago*, *Lotus*, *Arachis*, and *Cicer*) was intended to reduce artefacts of genome assembly by testing multiple accessions in single genera, and also to provide an opportunity to more confidently identify local evolutionary events, such as recent gene duplication and loss.

Nine *ACS* genes were identified in *M. truncatula*, *L. japonicus*, and *A. duranensis*, whereas eight *ACS* genes were found in *A. ipaensis* and the *Cicer* species (Table 1). Regarding gene nomenclature, we propose maintaining the *ACS* gene names given by van Zeijl *et al.* (2018, Preprint), as this study contains the largest representation of members of the *ACS* gene family published in legume plants. However, the original list of *MtACS* genes included *MtACS5* (Medtr5g011400; MtrunA17_Chr5g0398431) and *MtACS7* (Medtr6g463260; MtrunA17_Chr6g0475681), which are orthologues of *AtACS10* and *AtACS12*, respectively. In *A. thaliana*, these proteins were initially labelled as ACS but were later found to lack ACS activity (Yamagami *et al.*, 2003). Thus, we propose that *MtACS5* and *MtACS7* are Ala/Asp aminotransferases but not true *ACS* genes. Additionally, we could not detect transcripts for *MtACS4* in any of the RNA-seq databases analysed. Thus, we propose that *MtACS4* is likely a pseudogene. Using the *Medicago ACS* genes as a reference, we named the orthologues in the other legume species based on gene homology and the phylogenetic analysis explained below.

**Table 1.** Gene names and identities (IDs) of members of the *ACS* family in several legume species. In *M. truncatula* A17 genome version Mt5.0 the prefix ‘MtrunA17_’ was removed from the gene IDs. In *L. japonicus* Gifu, the prefix ‘LotjaGi’ was removed. Naming of the *ACS* genes follows that described in van Zeijl *et al.* (2018, Preprint) for the *M. truncatula ACS* family. *MtACS5* and *MtACS7* were excluded because they are likely not true ACS enzymes (Yamagami *et al.*, 2003). *MtACS4* is likely a pseudogene. The closest orthologues in other legume species are given the same name taking *M. truncatula* as a reference. Members were ordered based on the presence or absence of phosphorylation motifs in the C-terminal region of the predicted proteins

	Type I (MAPK ^*a*^ and CDPK ^*b*^ sites)				Type II (CDPK ^*b*^ site)		Type III (no predicted phosphorylation site)				
*Medicago truncatula*	*MtACS10*	*-*	*MtACS9*	*MtACS8*	*MtACS4*	*MtACS6*	*MtACS2*	*MtACS3*	*-*	*MtACS1a*	*MtACS1b*
A17 (Mt5.0)	Chr8g0387481		Chr8g0348351	Chr7g0248681	Chr3g0135251	Chr5g0400831	Chr4g0054371	Chr6g0488041		Chr8g0389311	Chr8g0389321
A17 (Mt4.0)	Medtr8g098930		Medtr8g028600	Medtr7g079080	Medtr3g103550	Medtr5g015020	Medtr4g097540	Medtr6g091760		Medtr8g101750	Medtr8g101820
R108	MtrG021904		MtrG036751	MtrG033286	MtrG015619	MtrG023047	MtrG039071	MtrG030296		MtrG022066	MtrG022068
***Lotus japonicus***	***LjACS10***	***LjACS11***	***LjACS9***	***LjACS8***	***LjACS4***	***LjACS6***	***LjACS2***	***LjACS3***	***-***	***LjACS1***	***-***
Gifu	4g1v0430100	2g1v0436200	3g1v0387700	1g1v0481200	1g1v0364000	2g1v0412200	4g1v0091800	2g1v0230900		4g1v0441500	
MG-20	Lj4g3v2990130	Lj2g3v2051190	-	Lj1g3v2766570	Lj1g3v1781070	Lj2g3v1982940	Lj0g3v0243559	Lj2g3v0909590		Lj4g3v3017360	
***Cicer arietinum***	***CaACS10***	***-***	***CaACS9***	***CaACS8***	***-***	***CaACS6***	***CaACS2***	***CaACS3a***	***CaACS3b***	***CaACS1***	*-*
ICCV 96029	Ca6g057900		Ca7g330700	Ca3g177400		Ca8g175800	Ca7g144700	Ca8g003800	Ca5g274400	Ca6g044400	
***Cicer echinospermum***	***CeACS10***	***-***	***CeACS9***	***CeACS8***	***-***	***CeACS6***	***CeACS2***	***CeACS3a***	***CeACS3b***	***CeACS1***	***-***
	Chr6:5406400-5408579		Chr7:57378871-57374714	Chr3:15472684-15476264		Chr8:49495396-49497273	Chr1:12915698-12914101	Chr8:1187060-1184343	Chr5:90016973-90018818	Chr6:4315148-4316731	
***Cicer reticulatum***	***CrACS10***	***-***	***CrACS9***	***CrACS8***	***-***	***CrACS6***	***CrACS2***	***CrACS3a***	***CrACS3b***	***CrACS1***	***-***
	Cr6g063300		Cr7g352700	Cr3g193600		Cr8g189700	Cr7g154500	STRG.79930	Cr5g308100	Cr6g049500	
***Arachis duranensis***	***AdACS10***	***AdACS11***	***AdACS9***	***AdACS8***	***AdACS4***	***AdACS6***	***AdACS2***	***AdACS3***	***-***	***AdACS1***	***-***
	XP_015955452	XP_015964337	XP_015947781	XP_015961831	XP_015935084	XP_015963701	XP_015958568	XP_052118284		XP_015955230	
***Arachis ipaensis***	***AiACS10***		***AiACS9***	***AiACS8***	***AiACS4***	***AiACS6***	***AiACS2***	***AiACS3***	***-***	***AiACS1***	***-***
	XP_016189469	-	XP_016186405	XP_016196741	XP_016165333	XP_016200622	XP_016174699	XP_016203825		XP_016193460	

^*a*^ MAPK, mitogen-activated protein kinase. ^*b*^ CDPK, calcium-dependent protein kinase.

To identify members of the *ACO* gene family, we used a similar reciprocal-BLAST approach using *Arabidopsis ACO* genes as queries (Table 2). Of the 14 genes annotated as ACO in *M. truncatula* genome version 5.0 (Mtv5.0; Pecrix *et al.*, 2018; https://medicago.toulouse.inra.fr/MtrunA17r5.0-ANR), only five were identified using reciprocal-BLAST. Analysis of conserved domains of the remaining seven genes annotated as *ACO* in the Mt5.0 genome version suggests that they belong to the broader 2-oxoglutarate and Fe(II)-dependent oxygenase superfamily but do not encode true ACO enzymes (https://www.ncbi.nlm.nih.gov/Structure/bwrpsb/bwrpsb.cgi; Wang *et al.*, 2023). In addition, two BLAST hits were found to correspond to the 5′ and 3′ fragments of a single *ACO* gene disrupted by a DNA transposon-like sequence, which were separately annotated as MtrunA17_Chr4g0075431 and MtrunA17_Chr4g0075471, respectively (*M. truncatula* genome version 5.0). These analyses suggest that the *M. truncatula* genome encodes five functional *ACO* genes and a sixth non-functional pseudogene. As this family has not been systematically analysed in legume plants in the literature, we named these genes in increasing order based on their location on the chromosomes in *M. truncatula*. Orthologues in the other legume species analysed in Table 2 were also named using the *M. truncatula* genes as a reference. The genome of *M. truncatula* and the three *Cicer* species analysed contained five *ACO* genes; six *ACO* genes were identified in the genome of *L. japonicus*, seven in *A. duranensis*, and eight in *A. ipaensis* (Table 2).

**Table 2.** Gene names and identities (IDs) of *ACO* gene family members in several legume species. In *M. truncatula* A17 genome version Mt5.0 the prefix ‘MtrunA17_’ was removed from the gene IDs. In *L. japonicus* Gifu, the prefix ‘LotjaGi’ was removed. Members were ordered based on the sequence of the functional motif R-X-S (Seo *et al*., 2004; Houben and Van de Poel, 2019)

	Type I (R-M-S motif)						Type II (R-L/I-S motif)	Type III (R-R-S motif)
*Medicago truncatula*	*MtACO1*	*MtACO2*	*-*	*-*	*MtACO4*	*-*	*MtACO5*	*MtACO3*
A17 (Mt5.0)	Chr2g0289341	Chr3g0121141			Chr5g0439011		Chr6g0488511	Chr3g0125201
A17 (Mt4.0)	Medtr2g025120	Medtr3g083370			Medtr5g085330		Medtr6g092620	Medtr3g088565
R108	MtrG006740	MtrG014334			MtrG026218		MtrG030346	MtrG014701
***Lotus japonicus***	***LjACO1***	***LjACO2***	***-***	***-***	***LjACO4***	***LjACO6***	***LjACO5***	***LjACO3***
Gifu	6g1v0289000	1g1v0066900			1g1v0067200	4g1v0010100	2g1v0232800	1g1v0266000
MG20	Lj6g3v1789900	Lj2g3v2879460			Lj0g3v0109219	Lj4g3v0120430	Lj2g3v0911850	Lj1g3v0884400
***Cicer arietinum***	***CaACO1***	***CaACO2***	***-***	***-***	***-***	***CaACO6***	***CaACO5***	***CaACO3***
ICCV 96029	Ca1g074700	Ca5g243300				Ca7g011100	Ca8g002800	Ca5g271800
***Cicer echinospermum***	***CeACO1***	***CeACO4***	***-***	***-***	***-***	***CeACO6***	***CeACO5***	***CeACO3***
	Chr1_19138489-19140282	Chr5_87202438-87200679				Chr1_922039-920440	Chr8_1247862-1249356	Chr5_89781373-89782790
***Cicer reticulatum***	***CrACO1***	***CrACO2***	***-***	***-***	***-***	***CrACO6***	***CrACO5***	***CrACO3***
	Cr1g079600	Cr5g275700				Cr7g011100	Cr8g003200	Cr5g305300
***Arachis duranensis***	***AdACO1***	***AdACO2a***	***AdACO2b***	***-***	***AdACO4***	***AdACO6***	***AdACO5***	***AdACO3***
	XP_015959035	XP_015973682	XP_015973680		XP_015973684	XP_015940837	XP_015966396	XP_015934593
***Arachis ipaensis***	***AiACO1***	***AiACO2a***	***AiACO2b***	***AiACO2c***	***AiACO4***	***AiACO6***	***AiACO5***	***AiACO3***
	XP_016197506	XP_016166204	XP_016166203	XP_016167479	XP_016166457	XP_016195518	XP_016203836	XP_016163470

### Protein alignment identifies conserved catalytic and regulatory domains in *Medicago* and *Lotus* ACS and ACO proteins

Analysis of the conserved protein domains of the ACS and ACO families has allowed the identification of functional and regulatory domains (Booker and DeLong, 2015). ACS proteins have conserved N-terminal regions containing seven sequence motifs required for ACS activity and a more variable C-terminus. The variable structure of the C-terminus is typically used to classify members of the ACS family into three distinct types of ACS enzymes, depending on the presence or absence of phosphorylation sites, based on the original classification in *A. thaliana* (Yamagami *et al.*, 2003). As such, type I ACSs have longer C-termini containing three Ser residues targeted by mitogen-activated protein kinases (MAPK) and a conserved calcium-dependent protein kinase (CDPK). However, type II ACSs contain only the CDPK site, whereas type III ACSs have shorter C-termini with no predicted phosphorylation sites (Tatsuki and Mori, 2001; Liu and Zhang, 2004; Chae and Kieber, 2005; Huang *et al.*, 2013). In *A. thaliana*, phosphorylation determines the stability of type-I ACS enzymes, which are the most labile ACSs (Joo *et al.*, 2008). Mutations in the C-terminal region of type II ACSs have been shown to improve protein stability and, thus, increase ethylene production (Vogel *et al.*, 1998; Chae *et al.*, 2003; Joo *et al.*, 2008). In contrast, the lack of predicted phosphorylation sites in type III ACSs suggests that this subgroup of ACS enzymes is more stable (Chae and Kieber, 2005).

**Fig. 1.**
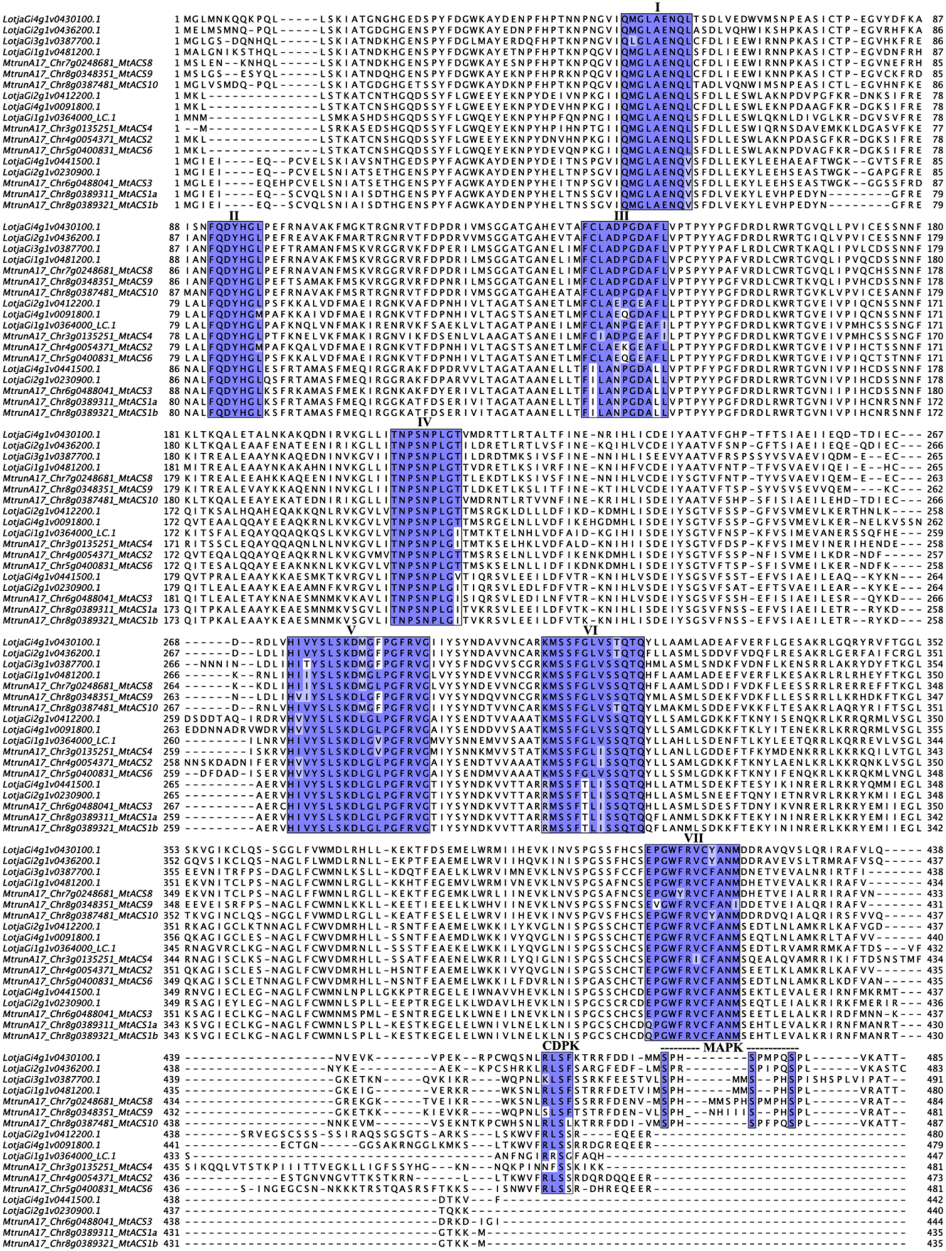
Alignment of the predicted protein sequences of the ACS family in the model legumes, *M. truncatula* A17 and *L. japonicus* Gifu. The conserved regions necessary for catalytic activity are highlighted in coloured boxes (numbered from I to VII). The presence of phosphorylation sites for calcium-dependent protein kinases (CDPKs) and mitogen-activated protein kinases (MAPKs) are also highlighted in the C-terminal regions.

Alignment of the predicted ACS proteins in *M. truncatula* and *L. japonicus* allowed for the identification of conserved residues required for catalytic activity (boxes I to VII in Fig. 1; Yamagami *et al.*, 2003). Furthermore, we were able to locate phosphorylation motifs in the N-terminal regions and thereby classify *ACS* genes according to the type I, II, or III enzymes that they encode (Fig. 1). *MtACS8*, *-9*, and *-10*, and *LjACS8*, *-9*, *-10*, and -*11* belong to type I (containing predicted MAPK and CDPK phosphorylation sites). *Medicago truncatula* and *L. japonicus ACS2*, *-4*, and *-6*, belong to type II (containing only a predicted CDPK site). *MtACS1a*, *-1b*, and *-3*, together with *LjACS1* and *-3* belong to the third category with no predicted phosphorylation site. By analogy to characterized enzymes in other plant species, the predicted phosphorylation sites in type I and II enzymes in legumes may regulate protein stability and the rate of ethylene synthesis *in planta*, though biochemical characterization will be required to test these predictions.

**Fig. 2.**
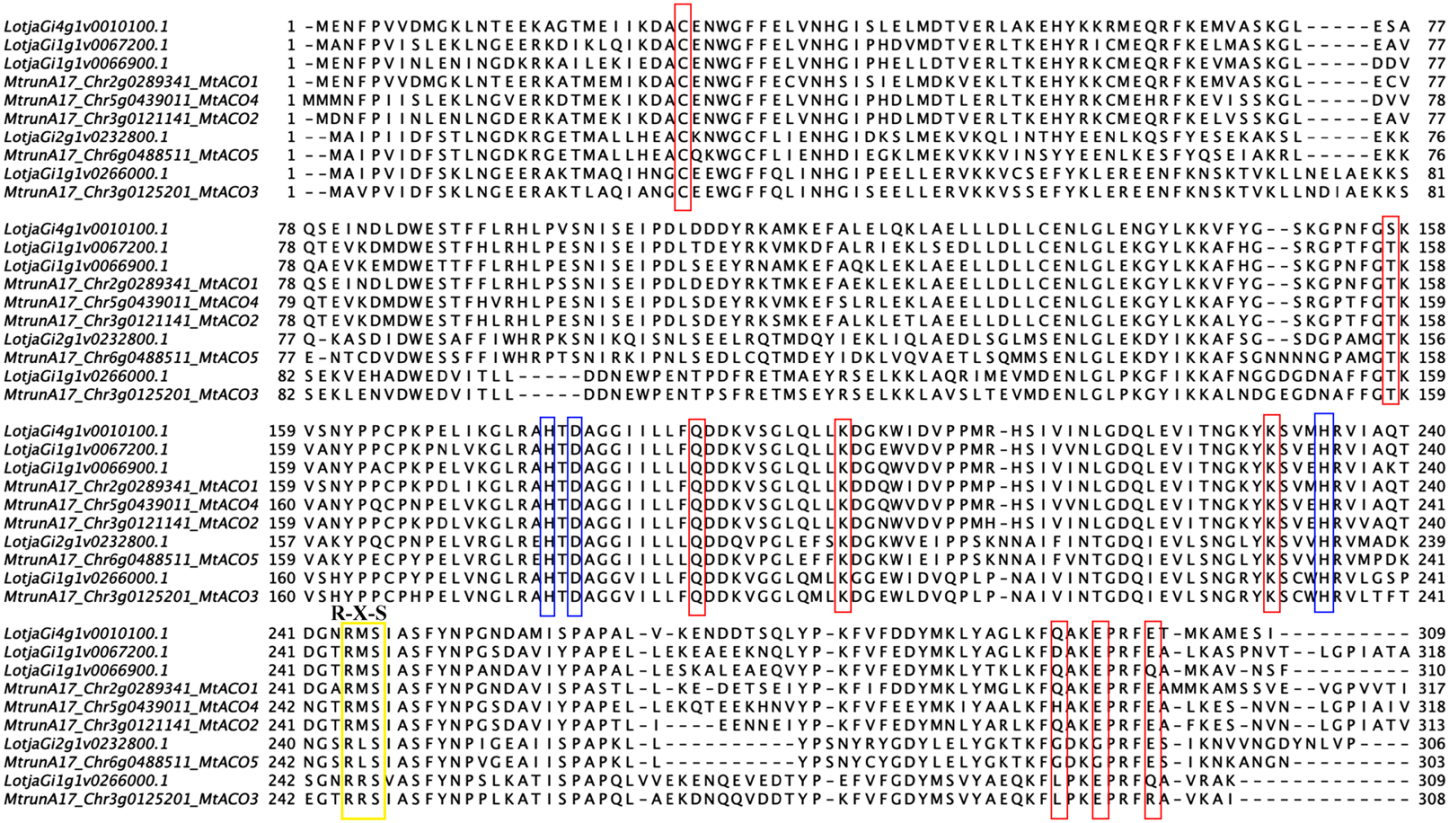
Alignment of predicted protein sequences of the ACO family in the model legumes *M. truncatula* A17 and *L. japonicus* Gifu. The conserved regions necessary for catalytic activity are highlighted in red boxes (Dilley *et al.*, 2013), and residues necessary for iron binding are highlighted in blue (Shaw *et al.*, 1996). The conserved R-X-S motif, which is required for ACO activity and sorts the family into three categories, is highlighted in yellow (Seo *et al*., 2004; Houben and Van de Poel, 2019).

Similar to the ACS family, several conserved residues have been described to be required for function in the ACO family (Fig. 2). Among these, we located an Fe(II)-binding motif, first described in apple (Shaw *et al.*, 1996), involving two His residues and the carboxylate group of a third residue, which can be either Asp or Glu. In *Medicago* and *Lotus*, we identified two His residues and an Asp residue in conserved positions, which have been shown to be critical for ACC binding (Zhang *et al.*, 2004; Martinez and Hausinger, 2015). ACO enzymes can be subclassified into three types based on the intermediate residue present in the dioxygenase-specific conserved R-X-S-motif at the C-terminus: type I, which contains an Arg-Met-Ser motif (R-M-S); type II, with an Arg-Lys/Ile-Ser motif (R-L/I-S); and type III, which contains an Arg-Arg-Ser motif (R-R-S; Houben and Van de Poel, 2019). This motif, involved in generating the reaction product during enzymatic catalysis (Seo *et al.*, 2004), is conserved across species and can also be identified in the *M. truncatula* and *L. japonicus* proteins. The majority of *ACO* genes found in the *M. truncatula* genome are type I enzymes (*MtACO1*, *-2*, and *-4*). There is only a single member for each of the type II (*MtACO5*) and type III (*MtACO3*) enzymes (Fig. 2). Similarly, in *L. japonicus*, the majority of enzymes are classified as type I (*LjACO1*, *-2*, *-4*, and *-6*), with only one member for each of a type II (*LjACO5*) and type III (*LjACO3*) enzyme.

### Phylogenetic analyses of the ACS and ACO families show legume-specific duplication events

From an evolutionary perspective, *ACS* homologues are found in the genome sequence of virtually all land plants sequenced, including non-spermatophytes, whereas *ACO* homologues appear only in the genomes of angiosperms and gymnosperms (Li *et al.*, 2018). It has been proposed that *ACOs* in angiosperms diverged from a common pre-gymnosperm ancestor, which, in turn, originated from an evolutionarily distant 2-oxoglutarate-dependent dioxygenase in algae (Houben and Van de Poel, 2019). To further support gene identification in legumes and assess phylogenetic relationships within angiosperms, we compiled ACO and ACS protein sequences from the legumes *M. truncatula* Jemalong A17, *L. japonicus* Gifu, *C. arietinum* ICCV96029, and *A. duranensis*, and the non-legumes *A. thaliana*, rice (*Oryza sativa* L.), tomato (*Lycopersicon esculentum* Mill.), and *Amborella trichopoda* Baill. *Amborella trichopoda* is the monotypic sister species to all other angiosperms (Albert *et al.*, 2013) and was used to root clades and to understand the origin of ancestral duplications.

**Fig. 3.**
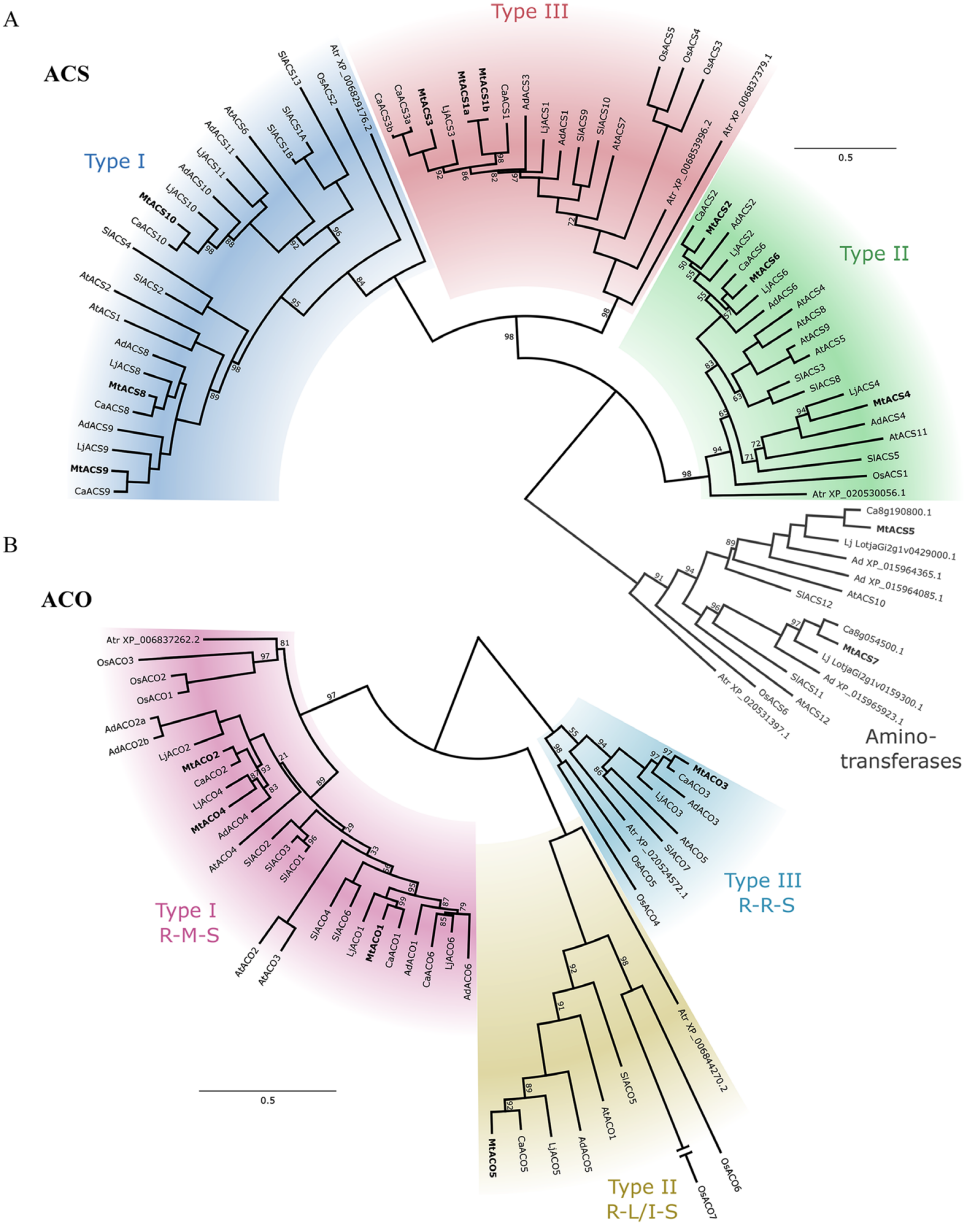
Phylogenetic analysis of ethylene biosynthesis genes across selected angiosperms. Phylogenetic trees of the *ACS* (A) and *ACO* (B) gene families from the legumes *Medicago truncatula* A17 (Mt; in bold), *Lotus japonicus* Gifu (Lj), *Cicer arietinum* ICCV 96029 (chickpea; Ca), and *Arachis duranensis* (peanut; Ad), and the non-legumes *Arabidopsis thaliana* (At), *Oryza sativa* (rice; Os), *Solanum lycopersicum* (tomato; Sl, and *Amborella trichopoda* (Atr), which was used to root clades and to understand the origin of ancestral duplications. Multiple sequence alignments were generated using MAFFT v.7.490 (Katoh and Standley, 2013) with algorithm selection, BLOSUM62 scoring matrix, a gap open penalty of 1.26, and an offset value of 0.123. Gene trees were reconstructed based on a Maximum Likelihood framework using IQ-TREE 2.2.2.3 (Minh *et al*., 2020) with automatic selection of the optimal model of sequence evolution (LG+T+G4 for both gene families) using ModelFinder and 100 non-parametric bootstraps to assess support. Colours indicate angiosperm orthogroups associated with phosphorylation and motif types; scale bars indicate 0.5 amino acid substitutions per site; numbers indicate bootstrap support values.

The *ACS* gene tree classifies *ACS* genes into three main orthologous groups (Fig. 3A), each containing members from all analysed species and each rooted by a single *ACS* gene from *A. trichopoda*. Thus, these three groups appear to pre-date the angiosperm radiation, while their radiation matches exactly with the presence (or absence) of MAPK and CDPK regulatory sites at the C-terminus of the proteins (type I/II/III ACS). Within the type I orthogroup, an eudicot-specific gene duplication event generated two paralogous groups. Each of these groups underwent subsequent legume-specific duplication. In the first group, the *ACS10* lineage is fully retained in the sampled legumes, while the *ACS11* lineage experienced loss in the cool season legume lineage (*Medicago* and *Cicer*). In the second group, the legume-specific duplication gave rise to the paralogous lineages *ACS9* and *ACS8*, each of which is fully retained across the sampled species. In the type II orthogroup, an eudicot-specific duplication gave rise to two paralogous groups. One group contains *ACS2* and *ACS6* that derive from a legume-specific duplication, while the second group contains *ACS4* within a single monophyletic eudicot clade. Although bootstrap support is low and gene placement is not fully reflective of known species relationships, the simplest interpretation of the *ACS2/ACS6* clade is that all orthologues are present in all species. Conversely, *ACS4* is missing from the *Cicer* lineages, which is supported by the absence of *ACS4* from all *Cicer* species with available complete genome data (https://cicer.legumeinfo.org/). Within the type III orthogroup, a legume-specific gene duplication generated two paralogous clades, containing *ACS1* and *ACS3*, with both *ACS1* and *ACS3* experiencing subsequent species-specific tandem duplication in *M. truncatula* and *Cicer*, respectively, leading to *MtACS1a* and *MtACS1b*, and *CaACS3a* and *CaACS3b.* Presumptive orthologues of *ACS1* and *ACS3* were inferred for both *Lotus* and *Arachis*; however, with the exception of *LjACS3*, their placement within the tree is ambiguous.

Similarly to the *ACS* family, *ACO* genes in legumes can be classified into three main clades, as previously observed in other plant species, including both angiosperms (Jafari *et al.*, 2013) and non-seed plants (Clouse and Carraro, 2014). The *ACO* gene tree derives from two ancient gene duplications, resulting in three angiosperm orthogroups that correspond to the three dioxygenase-specific R-X-S-motif at the C-terminus of the proteins (Fig. 3B). Within the type I orthogroup (motif R-M-S), a gene duplication at the base of the eudicots resulted in two paralogous groups, each of which experienced an additional legume-specific duplication, resulting in four legume clades. Each of these clades has a history of further species-specific duplications and losses, supported by symmetrical gene presence/absence in other genomes from the respective species (https://cicer.legumeinfo.org/). For example, in three of these four clades, *Medicago* retains a homologue (*MtACO1*, *MtACO2*, and *MtACO4*). Type II (R-L/I-S) and type III (R-R-S) orthogroups each contain a single *Medicago* orthologue (*MtACO5* and *MtACO3*, respectively). These genes in fact are single copy across all species analysed, with the exception of the monocot representative, in which each orthologue has undergone lineage-specific duplication.

### *ACS* and *ACO* gene expression show a finely-tuned spatiotemporal regulation at early symbiotic stages

**Fig. 4.**
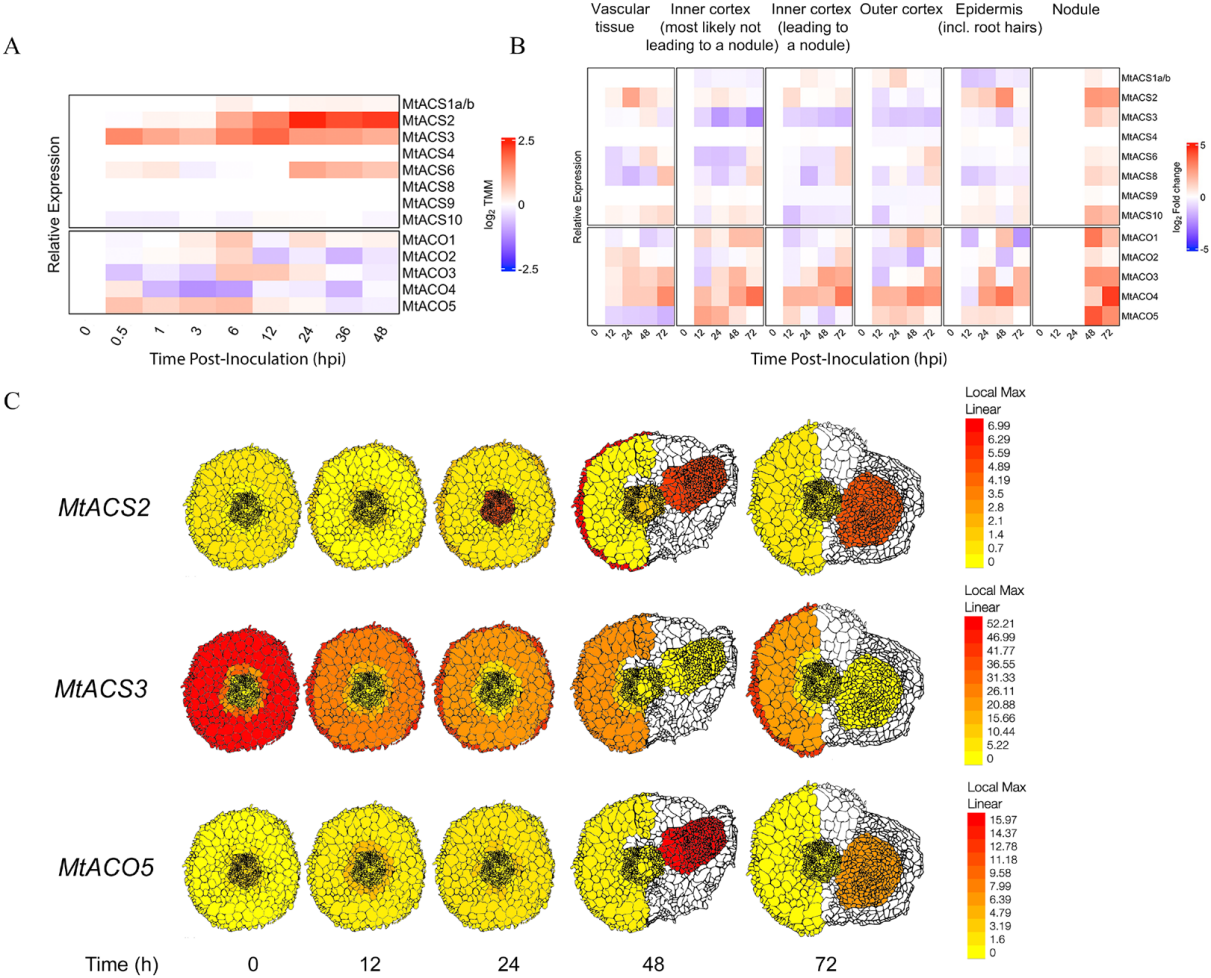
Representation of relative expression of members of the *ACS* and *ACO* gene families in *M. truncatula* A17 at different time points after inoculation with its microsymbiont. Expression data was extracted from (A) Larrainzar *et al*. (2015) and (B) Schnabel *et al.* (2023). (C) Spatio-temporal expression graphs of *MtACS2*, *MtACS3*, and *MtACO5* generated at ePlant (https://bar.utoronto.ca/eplant_medicago). Values are expressed in fragments per kilobase of transcript per million mapped reads (FPKM). Heatmaps depict log_2_-transformed relative Trimmed Mean of M-values (TMM) expression values (A) or log_2_-fold change (B) using time 0 h as a reference. hpi, hours post-inoculation. dpi, days post-inoculation.

With a confident set of *ACS* and *ACO* genes identified, we sought to understand and compare expression patterns of these genes during early nodulation. To do so, we focused on *M. truncatula*, for which we have access to an updated version of the RNA-seq dataset generated by Larrainzar *et al*. (2015), now remapped to the latest genome version (Mt5.0; Pecrix *et al.*, 2018). For the analysis, we considered *MtACS1a* and *MtACS1b* to be a single transcript source because the near-identical sequences preclude their separate analysis in RNA-seq datasets. From this dataset, we extracted data corresponding to the Jemalong A17 genotype from non-inoculated roots to roots at 48 h post-inoculation (hpi; Fig. 4A). Three *ACS* genes were found differentially expressed in A17 roots (log_2_ fold-change >1.5): *MtACS2*, *MtACS3*, and *MtACS6*. The expression pattern of the members of the *ACO* family varied across time but did not show differential expression. We also compiled single cell RNA-seq expression data from Schnabel *et al.* (2023), in which tissues from the maturation zone of *M. truncatula* roots were isolated using laser capture microdissection during a 72 hpi time course (Fig. 4B). In this dataset, two genes showed an increase in expression in root segments responding to rhizobia at time points 48–72 hpi: *MtACS2* and *MtACO5*. These genes were expressed in different root cell layers, particularly the epidermis and nodule primordium (Fig. 4C; generated at ePlant; https://bar.utoronto.ca/eplant_medicago; Waese *et al.*, 2017).

We additionally retrieved single-nucleus transcriptomic data of root cells treated with isolated Nod factors (Liu *et al.*, 2023). We attempted to collect information from other large-scale single-cell studies (Cervantes-Pérez *et al.*, 2022; Frank *et al.*, 2023), but were unable to retrieve data for the complete set of *ACS* and *ACO* genes. We did not include the transcription dataset generated in Schiessl *et al.* (2019) which did not match well with the Larrainzar *et al.* (2015) data because of the use of amino-ethoxy-vinyl-glycine (AVG) in the experimental design. AVG is a competitive inhibitor of the biosynthesis of ACC and likely promotes feedback regulation, complicating data interpretation. Single-nucleus expression data in response to Nod factors once more corroborated the differential response of *MtACS2* (>200 log_2_ fold-change) in epidermal root hair cells at 0.5 h post-Nod factor treatment (Liu *et al.*, 2023). *MtACO5* was not found differentially expressed in this dataset, although *MtACO2* and *MtACO4* were. This suggests that the symbiotic regulation of the expression of *MtACO5* occurs at later stages of nodule organogenesis (>48 hpi) and it is not mediated by Nod factors alone. *MtACS3* showed a complex expression pattern in the different datasets. In Larrainzar *et al*. (2015), *MtACS3* showed a transient increase in expression at 0.5 hpi and then at 12 hpi (Fig. 4A). Note that in this experiment whole roots were collected, so the final transcript count observed also includes other root regions not specifically involved in nodulation initiation. In contrast, single-cell analysis data indicated that this gene is down-regulated in the root cortex and epidermis layers upon inoculation, with a slight increase in expression in the nodule primordium (Fig. 4B). Data retrieved from Liu *et al*. (2023) showed increased *MtACS3* expression across the whole root (1.15 log_2_ fold-change), including the epidermis, at 0.5 h post-application of Nod factors, while expression declined at 24 h (–1.87 log_2_ fold-change). Unexpectedly, *MtACS6*, despite being up-regulated in the Larrainzar *et al*. (2015) dataset, was not differentially regulated in the two cell-type specific datasets.

Discrepancies between the different expression datasets can be explained by differences in experimental design, including factors such as type of treatment (isolated Nod factors versus rhizobia), specific tissues analysed (whole roots versus root fragments), and plant growth conditions (aeroponic cultures versus plates). Nevertheless, two general conclusions can be drawn. First, multiple *ACS* and *ACO* genes are redundantly expressed simultaneously in different root tissues, as shown most clearly for *MtACS2*, which is induced in response to both Nod factors and rhizobia (van Zeijl *et al.*, 2015; Liu *et al.*, 2023; Fig. 4). Second, expression patterns within these ethylene biosynthesis gene families are complex and dynamic, as shown for the case of *MtACS3* (Fig. 4C).

## Conclusions and perspectives

Here, we propose a unifying nomenclature and gene identification for members of the *ACS* and *ACO* gene families in the genomes of two model legumes, *M. truncatula* and *L. japonicus*, as well as the crops legumes chickpea and peanut. Although we did not test the biochemical properties of these enzymes, phylogenetic analyses, reciprocal-BLAST, and the identification of key conserved motifs for enzymatic activity and post-transcriptional regulation support the role of these genes as part of the ethylene biosynthesis pathway. Compilation of expression data from different available datasets in *M. truncatula* indicated that gene duplication was accompanied by a diversification of expression patterns, including several *MtACS* paralogues whose expression is primarily associated with the stages of early infection, particularly *MtACS2*. In *A. thaliana*, members of the *ACS* gene family exhibit a degree of tissue specificity, but also have overlapping expression patterns, with transcripts typically observed in hypocotyls, roots, abscission zones of siliques, and flowers (Tsuchisaka and Theologis, 2004; Bours *et al.*, 2015). Furthermore, ACS isoforms can form homo- and heterodimers with multiple possible functional combinations (Tsuchisaka and Theologis, 2004; Lin *et al.*, 2009). Combined reverse genetic and biochemical studies would be required to quantify the relative contribution of individual genes to ethylene production in legumes.

This meta-analysis provides a first step toward understanding the complexity of ethylene biosynthesis during nodule initiation in legumes. There are still questions that need to be addressed: can we differentiate the role of ethylene at early and later symbiotic stages? Can we identify the transcription factors involved in the symbiotic-dependent expression of ethylene biosynthesis genes? What are the effects of phosphorylation of predicted motifs on the different *ACS* and *ACO* proteins?

Besides the study of the *ACS* and *ACO* families, the precursor of ethylene, ACC, has emerged as a relevant signalling molecule during the past two decades. It has been shown to regulate key physiological processes in plants such as vegetative growth, cell division, and pollen tube development, among others (Xu *et al*., 2008; Tsang *et al.*, 2011; Van de Poel and Van Der Straeten, 2014; Vanderstraeten *et al*., 2019; Yin *et al*., 2019; Mou *et al*., 2020). When added exogenously, ACC is converted to ethylene through the action of ACO enzymes; thus, in legume nodulation studies, ACC is often used as a pharmacological inhibitor of nodulation, while its potential role as an endogenous regulatory molecule has not been studied. In *A. thaliana*, mutants in which all eight functional *ACS* genes were silenced allowed the identification of processes dependent on ACC per se, independently of ethylene biosynthesis (Tsuchisaka *et al*., 2009). A future challenge will be to generate multiple *ACS* or *ACO* gene mutants in legumes to be able to compare their responses to those of ethylene signalling mutants like *skl* (*ein2*). Alternatively, the effect of exogenous ACC treatment versus ethylene gas application may provide valuable insights into the role of this signalling molecule in legumes.

## Data Availability

No new data were generated or analysed in support of this research.
